# Anesthetic management of a patient with subclinical myasthenia gravis who underwent a thymectomy: a case report

**DOI:** 10.1186/s40981-022-00541-4

**Published:** 2022-07-15

**Authors:** Satoshi Uchida, Reiko Kudo, Daiki Takekawa, Kazuyoshi Hirota

**Affiliations:** Department of Anesthesiology, Hirosaki University Graduate School of Medicine, 5 Zaifu-cho, Hirosaki, 036-8562 Japan

**Keywords:** Subclinical myasthenia gravis, Anti-acetylcholine receptor antibodies, Extended thymectomy

## Abstract

**Background:**

Some individuals with subclinical myasthenia gravis (MG) are positive for serum anti-acetylcholine receptor antibodies, without neurological symptoms. There are no anesthetic management guidelines for subclinical MG. We report the anesthetic management of a patient with subclinical MG who underwent a thymectomy.

**Case presentation:**

A 57-year-old female with subclinical MG was scheduled for an extended thymectomy. Anesthesia was induced and maintained with mainly propofol and remifentanil. We administrated the minimum amount of rocuronium with reference to train-of-four (TOF) monitoring when a neuromuscular relaxant is needed. Although the prolonged effect of rocuronium was observed, the TOF ratio had already recovered to 100% before the tracheal extubation. Postoperative analgesia was performed by a continuous epidural infusion of levobupivacaine.

**Conclusion:**

We reported the anesthetic management of a patient with subclinical MG who underwent a thymectomy. Further research is necessary to clarify subclinical MG patients' sensitivity to rocuronium.

## Background

Myasthenia gravis (MG) is an autoimmune disorder typically characterized by muscle weakness. Anti-acetylcholine receptor (AChR) antibodies are detected in the most individuals who have MG. It has been reported that patients with MG are sensitive to non-depolarizing neuromuscular blocking agents (NMBAs) [1]. In addition, a myasthenic crisis after a thymectomy is a life-threatening complication, i.e., a rapid worsening of MG symptoms; a meta-analysis reported that the prevalence of post-thymectomy myasthenic crisis is 17.5% [2]. The perioperative management of patients with MG is thus challenging.

There are few patients with subclinical MG who is positive for serum anti-AChR antibodies without neurological symptoms [3]. However, because of the low prevalence, our PubMed search could not find any articles that describe the anesthetic management of the patients with subclinical MG, and the sensitivity of these patients to non-depolarizing NMBAs is not yet known. We report the anesthetic management of a patient with subclinical MG who underwent a thymectomy.

## Case presentation

We obtained a written informed consent from the patient for the publication of this case report.

A 56-year-old female (height, 158 cm; weight 77 kg) was scheduled for an extended thymectomy for invasive thymoma. She had a history of caesarean section and was not taking any medications. Although the anti-AChR antibody was seropositive (0.4 nmol/L, reference range ≤ 0.2 nmol/L), no neurological findings (e.g., muscular fatigue, double vision, bilateral ptosis) were identified at any time point. She also had no abnormal physical examination data, including spirometry.

Epidural catheter was already inserted via the Th 5–6 intervertebral space a day before surgery. On the morning of the thymectomy, the patient was administrated 75 mg of roxatidine as anesthetic premedication without other routine premedications (e.g., benzodiazepines) in order to avoid deep sedation. Anesthesia was induced with 80 mg of propofol, 20 mg of ketamine, and 0.3 μg/kg/min of remifentanil. An electromyographic module (AF-101P™, Nihon Kohden, Tokyo) was attached to monitor neuromuscular blockade and was calibrated to determine stimulus intensity before the administration of rocuronium. The left ulnar nerve was stimulated at the wrist to monitor the abductor digiti minimi muscle and the baseline train-of-four ratio (TOFR) was 100%. We administrated 5 mg of rocuronium to confirm sensitivity, and the TOFR decreased to 66% in 2 min. We administrated a total of 20 mg (0.26 mg/kg) of rocuronium in divided doses, and the train-of-four count (TOFC) became 0 at approximately 4 min from the first administration. A double-lumen endobronchial tube was intubated, without complications.

**Fig. 1:**
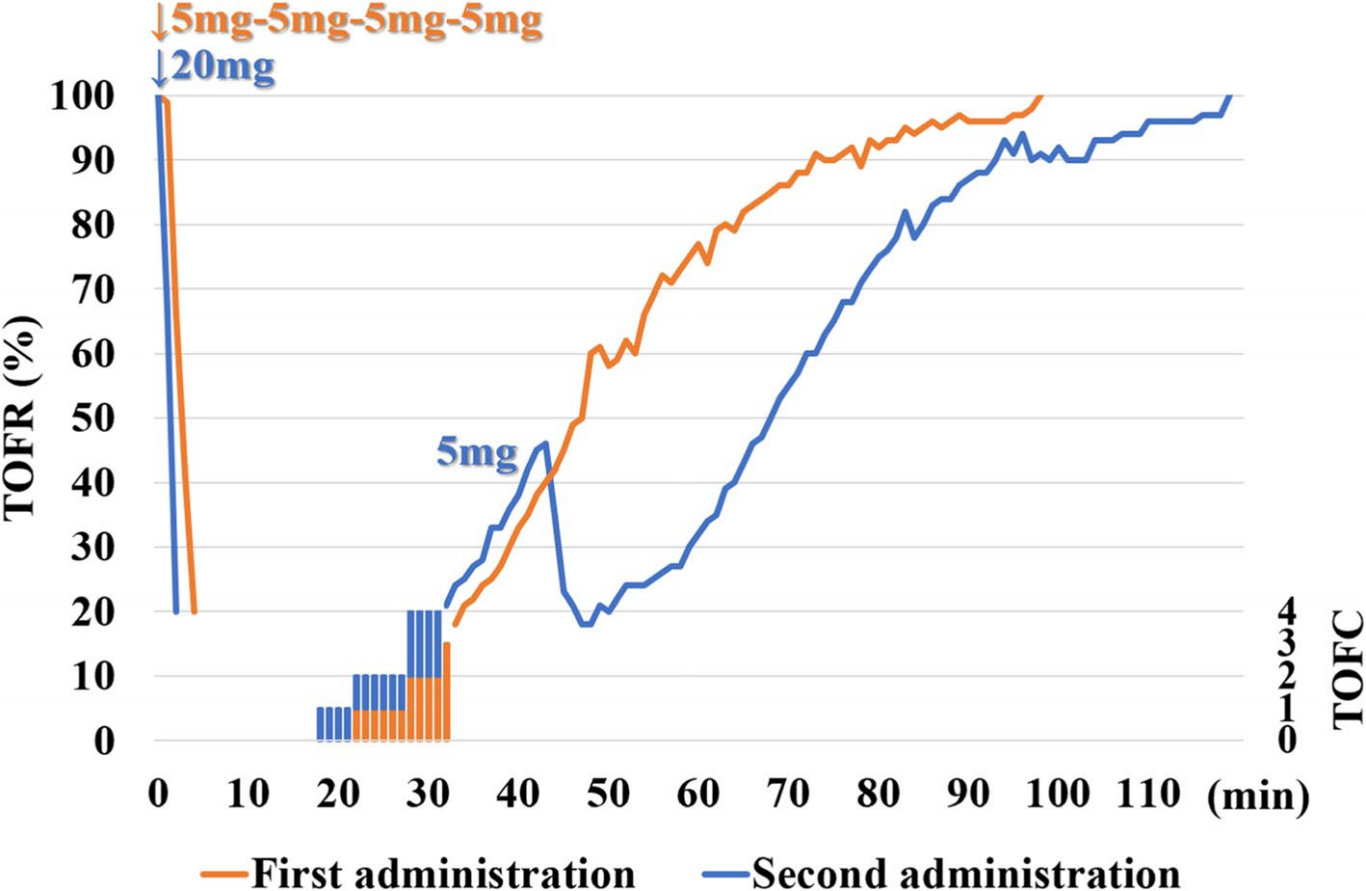
Time course of train-of-four counts and its ratio after administration of rocuronium. Rocuronium 20 mg in four divided doses was administered before tracheal intubation (first administration, orange line), 20 mg bolus and additional 5 mg were administered during surgery (second administration, blue line). TOF: train-of-four, TOFR: train-of-four ratio, TOFC: train-of-four count

Before the skin incision, 6 mL of 0.25% levobupivacaine and 2 mg of morphine were infused from an epidural catheter. Anesthesia was maintained with 4 mg/kg/h of propofol and 0.3–0.4 μg/kg/min of remifentanil. The TOFR gradually increased to 25%, 75%, and 90% at 37 min, 59 min, and 73 min, respectively, with no additional infusion of rocuronium. Approximately 4 h later, we administrated another 20 mg of rocuronium to prevent fatal complications caused by body movement; we did so because the patient's thymoma invaded the superior vena cava, and a partial resection of the superior vena cava was necessary. The TOFC immediately decreased to 0 (within nearly 1.5 min), and it took 34 min to reach TOFR 25%. We added 5 mg of rocuronium once to keep the TOFR low until the partial resection of the superior vena cava was performed (Fig. 1). Moreover, since the thymoma also invaded the right lung middle lobe and right phrenic nerve, a partial resection of right lung middle lobe was added and the right phrenic nerve was sacrificed. The surgery was completed without body movement and lasted 5 h 38 min, with 620 g of the total blood loss. The depth of anesthesia was monitored using the Bispectral Index (BIS) and the dose of anesthetic was adjusted appropriately so that the BIS was in the range of 40–60. Body temperature during anesthesia was controlled above 36°. Postoperative analgesia was provided by a continuous epidural infusion of 0.25% levobupivacaine at 2 mL/h.

The patient emerged from anesthesia clearly and had tachypnea (respiratory rate 30/min, tidal volume 300–350 mL), but did not complain of dyspnea or pain. The tracheal tube was extubated without an administration of sugammadex, because the TOFR had already recovered to 100%. After the patient's tracheal extubation, her respiratory rate decreased to 25/min. She was then transferred to the intensive care unit. On the following morning, her respiratory rate had decreased to 20/min and she did not complain of dyspnea. The postoperative course was uneventful with no respiratory complications (such as respiratory depression) and progression of muscle weakness. The patient was discharged on the 9th postoperative day.

## Discussion

We present a case of subclinical MG in thymoma under general anesthesia. The extended thymectomy was successfully managed with total intravenous anesthesia and epidural anesthesia. We administrated the minimum amount of rocuronium with reference to TOF monitoring when a neuromuscular relaxant is needed. Postoperative analgesia was provided by a continuous epidural infusion of levobupivacaine. There are no guidelines for the anesthetic management of patients with subclinical MG. However, as 91% of patients with subclinical MG were reported to develop clinical MG within 6 years after a thymectomy [3], we managed this patient's surgery in accord with the anesthetic management of patients with MG.

Generally, inhalation anesthesia has the effect of inhibiting neuromuscular transmission, and the inhibitory effects were reported to be more prominent in patients with MG [4]. We thus chose total intravenous anesthesia using propofol and remifentanil (which do not affect neuromuscular transmission) to assess the exact effects of rocuronium using TOF monitoring.

Individuals with some types of neuromuscular disorders are known to be markedly sensitive to non-depolarizing NMBAs [5]. Although the sensitivity of patients with subclinical MG to non-depolarizing NMBAs has not been clarified, patients with MG were reported to be sensitive to non-depolarizing NMBAs [1]. The successful anesthetic management of patients with MG without non-depolarizing NMBAs has been reported [6]. However, tracheal intubation without NMBAs can lead to an increased risk of difficult tracheal intubation and upper airway discomfort or injury [7]. The successful use of sugammadex to reverse rocuronium in patients with MG was reported [8], but two cases in which a neuromuscular block induced by rocuronium could not be reversed with sugammadex were also described [9, 10]. In our case, difficult tracheal intubation was expected because her body mass index was 30.8 kg/m^2^ and her preoperative Mallampati scale was III. We therefore administrated the minimum amount of rocuronium with reference to TOF monitoring when we performed her tracheal intubation.

In a clinical trial of rocuronium in Japanese patients, at the first administration of 0.3 mg/kg of rocuronium, the onset time (defined as achieving a TOFC 0) was 271.5 ± 73.6 s (mean ± standard deviation), and clinical duration (defined as 25% recovery of T1) was 17.4 ± 8.9 min (mean±standard deviation) [11]. For our patient, we administrated 20 mg (0.26 mg/kg) of rocuronium twice under a 100% TOFR. At anesthetic induction, the onset time and clinical duration were 4.5 min and 37 min, but it should be considered slow and divided administration. The onset time and clinical duration at the second administration of rocuronium (20 mg) that was administrated at once and not divided were 1.5 min and 34 min, respectively (Table 1). The onset time was shorter and clinical duration was longer compared to the reference range as well as the first administration (Table 1). The patient thus exhibited a prolonged effect of rocuronium, which indicates that some patients with subclinical MG may be hypersensitive to non-depolarizing NMBAs.

**Table 1:** Comparison of onset time and clinical duration

	Dose of rocuronium	Onset time	Clinical duration
**Refferance range (11)**	**0.3 mg/kg**	**271.5 ± 73.6 s**	**17.4 ± 8.9 min**
**First administration**	**0.26 mg/kg**	**4.5 min**	**37 min**
**Second administration**	**0.26 mg/kg**	**1.5 min**	**34 min**

Data are presented as (mean ± standard deviation)

For postoperative analgesia in patients with MG, epidural anesthesia is recommended in order to reduce opioid use and to avoid postoperative respiratory complications [12]. In our case, the use of epidural anesthesia avoided the need for the administration of opioids for postoperative analgesia. Although the patient had tachypnea due to phrenic nerve palsy, her emergence from the anesthesia was clear and without residual effects of anesthetics, including muscle relaxation and premedication.

## Conclusion

We experienced a case of subclinical MG in thymoma under general anesthesia. Although the sensitivity of patients with subclinical MG to non-depolarizing NMBAs is not yet known, a prolonged effect of rocuronium was observed in this patient. Further research is necessary to clarify the sensitivity to non-depolarizing NMBAs in patients with subclinical MG.

## Data Availability

Not applicable.

## Abbreviations

MG: Myasthenia gravis
AChR: Anti-acetylcholine receptor
NMBA: Neuromuscular blocking agent
TOFR: Train-of-four ratio
TOFC: Train-of-four count
BIS: Bispectral Index
